# Effect of Retirement on Alcohol Consumption: Longitudinal Evidence from the French Gazel Cohort Study

**DOI:** 10.1371/journal.pone.0026531

**Published:** 2011-10-20

**Authors:** Marie Zins, Alice Guéguen, Mika Kivimaki, Archana Singh-Manoux, Annette Leclerc, Jussi Vahtera, Hugo Westerlund, Jane E. Ferrie, Marcel Goldberg

**Affiliations:** 1 Inserm U1018 Epidemiology of Occupational and Social Determinants of Health - Centre for Research in Epidemiology and Population Health, Vilejuif, France; 2 Versailles-Saint Quentin University, Versailles, France; 3 Department of Epidemiology and Public Health, UCL, London, United Kingdom; 4 Finnish Institute of Occupational Health, Helsinki, Finland; 5 Department of Public Health, University of Turku, Turku, Finland; 6 Stress Research Institute, Stockholm University, Stockholm, Sweden; 7 Department of Epidemiology and Public Health, University of Helsinki, Helsinki, Finland; Federal University of Rio de Janeiro, Brazil

## Abstract

**Background:**

Little is known about the effect of retirement on alcohol consumption. The objectives were to examine changes in alcohol consumption following retirement, and whether these patterns differ by gender and socioeconomic status.

**Methods and Findings:**

We assessed alcohol consumption annually from 5 years before to 5 years after retirement among 10,023 men and 2,361 women of the French Gazel study. Data were analyzed separately for men and women, using repeated-measures logistic regression analysis with generalized estimating equations. Five years prior to retirement, the prevalence of heavy drinking was about 16% among men, and not patterned by socioeconomic status. Among women, this prevalence was 19.5% in managers, 14.7% in intermediate occupations, and 12.8% in clerical workers. Around retirement, the estimated prevalence of heavy drinking increased in both sexes. In men, this increase was 3.1 percentage points for managers, 3.2 in intermediate occupations, 4.6 in clerical workers, and 1.3 in manual workers. In women, this increase was 6.6 percentage points among managers, 4.3 in intermediate occupations, and 3.3 among clerical workers. In men the increase around retirement was followed by a decrease over the following four years, not significant among manual workers; among women such a decrease was also observed in the non-managerial occupations. It is difficult to assess the extent to which the results observed in this cohort would hold for other working populations, other conditions of employment, or in other cultural settings. A plausible explanation for the increase in heavy drinking around retirement could be that increased leisure time after retirement provides more opportunities for drinking, and not having to work during the day after may decrease constraints on drinking.

**Conclusions:**

Our findings of increased consumption around retirement suggest that information about negative effects of alcohol consumption should be included in pre-retirement planning programs.

## Introduction

In developed countries, disorders related to alcohol abuse are among the 10 leading contributors to the burden of disease and the fifth most important risk factor for loss of healthy life [1]. Due to physiological, biological or social changes related to ageing and increased prevalence of morbidity, older persons are at a greater risk of developing health problems as a consequence of excessive alcohol consumption [2]. Furthermore, as people live longer, the absolute number of older adults who continue to drink increases and the number of senior adults with problem drinking is likely to become an increasingly important public health issue.

The preretirement period may constitute a time of investment in healthy lifestyles for successful ageing, and could be a key period for preventive actions concerning alcohol consumption [3], [4]. However, little is known about the effect of retirement on alcohol consumption. Perreira and Sloan reported an increase in habitual consumption in relation to retirement using data from the Health and Retirement Study [5]. Brennan et al. conducted a 10-year longitudinal study comparing retirees and non-retirees, but did not examine the role of the retirement itself on alcohol consumption [6]. Other longitudinal studies suggest that there is little or no difference in patterns of alcohol drinking between retirees and non-retirees [7], [8]. These mixed findings suggest that the impact of retirement on alcohol consumption may be complex; long-term follow-up and repeated measures of alcohol consumption may therefore be needed. To date, no published evidence is available on trajectories of changes in alcohol consumption from pre- to post-retirement over an extended time period.

The aim of this study is to explore the effect of retirement on alcohol consumption using a longitudinal design with annual self-reported measures of alcohol intake from 5 years before to 5 years after retirement. The study is set in France, one of the European countries where alcohol consumption is highest, and where mortality from alcohol-related causes is 30% above the European average. It is well established that consumption patterns differ as a function of gender, there are also large socioeconomic differences in alcohol consumption that contribute to socioeconomic differences in premature mortality in France [9]. For these reasons, we stratified the analyses by sex and examined the impact of socioeconomic status (SES) on patterns of alcohol consumption around retirement.

## Methods

### Ethics Statement

The study protocol, including a written consent of the cohort participants, was approved by the French authority for data confidentiality (*Commission Nationale Informatique et Liberté #* 105728) and by the Ethics Evaluation Committee of INSERM (IRB0000388, FWA00005831).

### Study population

The Gazel cohort was established in 1989 and comprises employees of the French national gas and electricity company: Electricité de France-Gaz de France (EDF-GDF) [10], [11]. At baseline, 20 625 employees (73% men), aged 35–50, gave consent to participate. EDF-GDF employees have a status similar to that of civil servant status that guarantees job stability and opportunities for occupational mobility. Typically, employees are hired when they are in their 20 s and stay with the company until retirement. Pensions for retirees are paid by the company, which also records reasons for retirement such as disability and long-standing illness. Because of these characteristics, study follow-up is very thorough and few subjects are lost to follow-up.

### Data on retirement

Company data on retirement are comprehensive and accurate; less than 1% of the participants were lost to follow-up since study inception in 1989 [12], [13]. Statutory age of retirement is between 55 and 60 years depending on type of job: the longer an employee has worked in a blue collar job, the earlier s/he is allowed to retire. Although partial retirement is rare, retirement can, in some cases, occur before the age of 55. Retirement for health reasons can be granted in the event of long-standing illness or disability. Illness or disability claims are validated by the Social Security Department of EDF-GDF. From the company records, we obtained data on statutory retirement as well as retirement due to long-standing illness or disability. Only subjects who retired on a statutory basis were included in this study.

### Alcohol consumption

Data on health, lifestyle, alcohol consumption, and other characteristics of the Gazel participants were collected by annual surveys using a mailed self-administered questionnaire. Alcohol consumption was assessed every year from January 1992 to January 2007, using a validated instrument [14] comprised of the following questions: “*Have you consumed any wine (beer, cider, spirits) over the past week? If yes, what was the maximum quantity per day (number of glasses)? On how many days during the past week did you drink wine (beer, cider, spirits)?*” For each type of beverage, the volunteers checked a box under a drawing representing a standard drink.

We computed the number of drinks consumed weekly, and heavy alcohol consumers were defined as men (women) reporting at least 28 (14) drinks over a one-week period. These levels of drinking are considered to be a health risk by the World Health Organisation and the US National Institute on Alcohol Abuse and Alcoholism [2], [15]. We used annual measurements collected over a 10-year time window ranging from the 5th year preceding retirement to the 5th year after retirement.

### Pre-retirement covariates

#### Demographic factors

Men were grouped into 2 birth cohorts; 1939–1943 and 1944–1948. As women in GAZEL are somewhat younger, a third category, 1949–1953 was added.

The marker of SES used in this study was occupational position which was derived from EDF-GDF records and divided into four groups based on categorizations from the French National Statistics Institute, INSEE: managers, intermediate (technical staff, line managers, and administrative professionals), clerical workers, and manual workers [16].

We chose to use occupational grade at 35 to measure occupational position rather than at study inception or at retirement for all participants because of the high upward mobility of the cohort across the follow-up and a tendency for individuals to be promoted just before retirement; thus, a mid-career measure at an age where lifestyle habits are usually stabilized captures early upward mobility without being artificially inflated [17].

### Statistical methods

**Figure 1:**
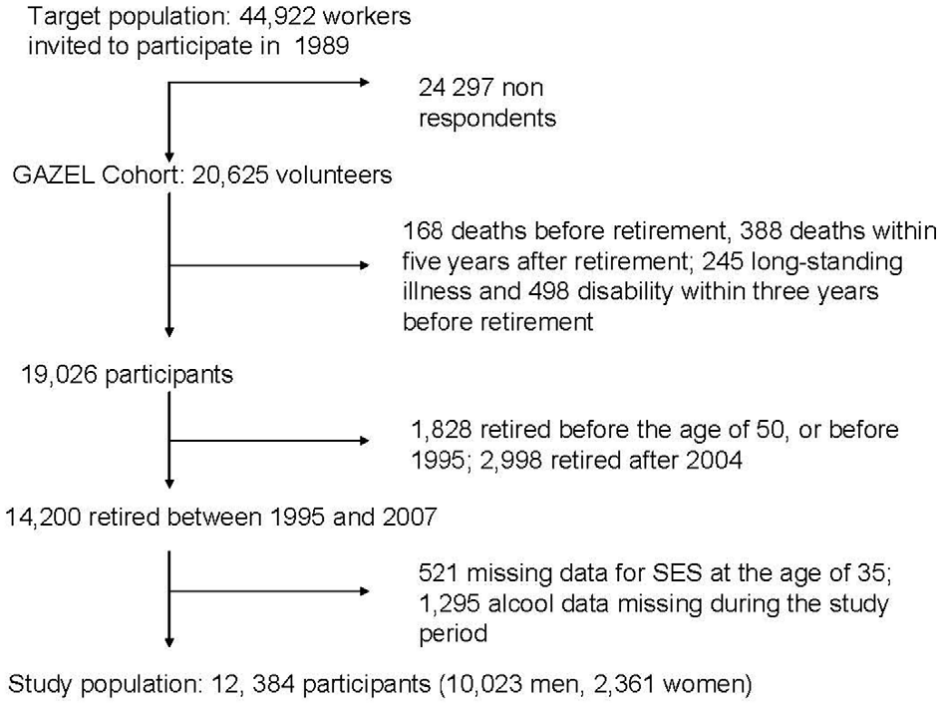
Flowchart describing the selection of participants in the study.

Participants in the current study were followed-up from 1992 to 2007. Because we wanted to examine changes in alcohol consumption over a 11-year window with data pre- and post-retirement, we included only those who had retired between 1995 and 2004. As shown in figure 1, we excluded: participants who died before retirement (n = 168); those who died within five years after retirement (n = 388); those who had retired due to a long-standing illness (n = 245) or disability (n = 498) within three years prior to statutory retirement; and those who retired before the age of 50 or before 1995 (n = 1828) or after 2004 (n = 2998). We also excluded Gazel participants for whom data on socioeconomic status at age 35 (n = 521) were missing, or who did not report alcohol consumption at least once in the five years before retirement and at least once after retirement (n = 1295). Thus, the analytic sample included 12 384 employees (10 023 men and 2361 women).

The analyses were conducted separately for men and women. We used Generalised Estimating Equations (GEE) logistic regression for binary data in order to take into account the correlation between repeat observations for the same subject [18]. Coefficients from these models can be interpreted as excess risk.

**Figure 2:**
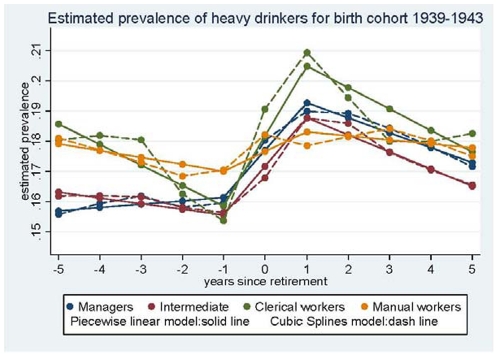
Estimated prevalence of heavy drinkers - Men.

**Figure 3:**
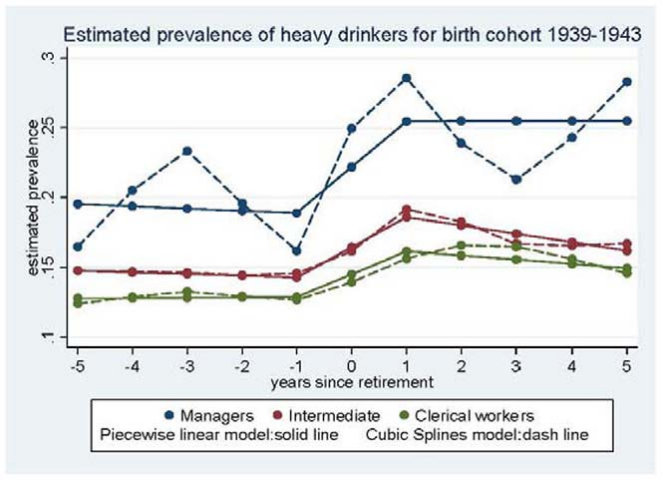
Estimated prevalence of heavy drinkers - Women.

In the first step, the trajectory of the proportion of heavy drinkers was modelled using cubic splines with 7 knots [19]. As these proportions differed according to SES and birth cohort (1939–1943, 1944–1948, and 1949–1953 in women), the models were run for each SES category and birth cohort. The proportion of heavy drinkers was lower in the younger birth cohorts. However, as trajectories of alcohol consumption over retirement did not vary (data not shown), the birth-cohorts were combined in further analyses. Figures 2 and 3 present the results for men and women respectively, for the 1939–1943 birth cohort (results were similar for the other birth cohorts). These preliminary analyses led us to model the trajectory of the proportion of heavy drinkers with a piecewise linear regression with inflection points at −1 year and at +1 year of retirement.

The analysis was based on a 11-year observation window with the year of retirement as year 0 and a 5-year observation period both before (years −5 to −1) and after retirement (years +1 to +5). The first model included the main effects of interest and the following interaction terms: “year × SES category”, “birth cohort × year”, “birth cohort × SES category”, and “birth cohort × year × SES category”. As the interactions with the birth cohorts were non-significant, the model reported here includes the following covariates: time window, birth cohort, SES category and the interaction “year × SES category”. This model was used to estimate: (a) the proportion of heavy drinkers at different times in relation to retirement (5 years and 1 year before, 1 year and 5 years after), according to SES category and birth cohort; (b) the change in the percentage of heavy drinkers according to SES category, in each time window (−5, −1), (−1, 1) and (1, 5).

We used Wald's test to examine the change in slope at −1 and +1 year of retirement for each SES category, to compare SES categories in relation to the proportion of heavy drinkers, and changes over time in these categories.

One or more observations may be missing for some participants included in the analytic sample, either due to questionnaire non-response, or to the design of the study. For instance, a cohort member who retired in 1995 may not have alcohol consumption data for the entire time window (−5 years, +5 years), even if he completed all the yearly questionnaires from 1992 to 2007. In order to assess the potential biases introduced by missing data, we compared the results of the GEE analyses for all 10 023 men (2361 women) to those for a sub-sample including only participants with no missing data. The analyses were performed using Stata (version 10). For longitudinal modeling, the xtgee procedure was used.

## Results

Table 1 shows the characteristics of the study participants. Between 1995 and 2004 10 023 men and 2 361 women retired; the largest group in both sexes was those in the intermediate SES group (59% and 57% of the total workforce respectively). Female manual workers were excluded from further analyses because of small numbers (n = 14). The mean age at retirement was 55.1±2.0 for men and 54.9±2.4 for women, but it varied between SES groups. 72% of the men and 64% of the women had retired by age 55, and more than 99% by age 60.

**Table 1:** Characteristics of the participants.

	Men (n = 10 023)				Women (n = 2 361)*		
	Manager(High SES)	Intermediate	Clerical	Manual(Low SES)	Manager(High SES)	Intermediate	Clerical(Low SES)
N	1 585	5 908	561	1 969	96	1 352	900
Birth cohorts n (%)							
1939–1943	861(8.6%)	2 097 (20.92)	280 (2.8)	704 (7.0)	44 (1.9)	395 (16.8)	325(13.8)
1944–1948	724(7.4%)	3 811 (38.02)	281 (2.8)	1 265(12.6)	35 (1.5)	711 (30.3)	453 (19.3)
1949–1953					17 (0.07)	246 (10.5)	118 (5.0)
Mean age (SD) at retirement	57.0 (2.3)	54.7 (1.7)	55.6 (1.9)	54.5 (1.4)	56.0 (3.0)	54.6 (2.3)	55.2 (2.4)
Range	50–63	50–61	50–61	50–60	50–61	50–61	50–61

*14 manual workers excluded.

The longitudinal analysis using repeated measures of alcohol consumption showed an increase in the proportion of heavy drinkers around the period of retirement among both men and women. Among men, this increase observed in all SES categories except manual workers, was temporary and was followed by a decrease (Figure 2); however, among manual workers, changes were small and the postretirement decrease was non significant. Among women managers the increase in the proportion of heavy drinkers remained unchanged during the entire post-retirement observation period; the decrease in alcohol consumption was significant in the intermediate SES category, but not in the clerical group (Figure 3).

### Trajectories of heavy drinking in men

The differences in the estimated proportions of heavy drinkers between the SES categories were small and not statistically significant at any period in relation to retirement (table 2). Table 3 reports the change in the estimated proportion according to SES and to the different periods around retirement. In the pre-retirement period (5 years to 1 year before retirement) there was no significant change in the proportion of participants who were heavy drinkers in any SES category, although there was a tendency towards a decrease among clerical workers. Around retirement (from 1 year before to 1 year after retirement), the percentage of participants who were heavy drinkers increased in all four SES categories. The increase was 3.1% for managers, 3.2% for intermediate occupations, and 4.6% for clerical workers. The only exception was manual workers, among whom the 1.3% increase was not significant. The tests for change in slope between the slope from −5 to −1 years from before retirement, to the slope from +1 to +5 years to after retirement were statistically significant (p<0.05) for all SES groups, except for manual workers (data not shown). There was an indication that the rate of increase (slope) was different between SES groups (p 0.07). Compared to the manual workers, the slope was steeper for clerical workers (test of interaction: p = 0.03), intermediate occupations (p = 0.02), and managers (p = 0.08) (data not shown). During the post-retirement period (from 1 to 5 years after retirement), there was a significant decrease in the proportion of heavy drinkers: −2.0% for managers, −2.4% for intermediate occupations and a strong indication, −2.8%, among clerical workers (p-value = 0.07), but no decrease was observed for manual workers. The tests for change in slope between −1 to +1 from retirement, to the slope from +1 to +5 after retirement were statistically significant for all SES categories except manual workers (results not shown).

**Table 2:** Proportion (95% confidence intervals (95% CI)) of heavy drinkers in relation to retirement, by SES in men (from general estimation equation models).

SES	5 years before	1 year before	1 year after	5 years after
	% (95% CI)	% (95% CI)	% (95% CI)	% (95% CI)
Managers	14.8 (13.1 to 16.5)	15.2 (13.6 to 16.9)	18.4 (16.6 to 20.2)	16.4 (14.6–18.2)
Intermediate	15.1 (14.1 to16.0)	14.3 (13.4 to 15.1)	17.5 (16.6 to 18.4)	15.3 (14.3–16.2)
Clerical workers	17.6 (14.4 to 20.8)	14.9 (12.0 to 17.8)	19.5 (16.3 to 22.7)	16.7 (13.5 to 19.9)
Manual workers	16.7 (15.0 to 18.4)	15.7 (14.2 to 17.3)	17.1 (15.4 to 18.7)	16.5 (14.9 to 18.2)
Difference between SES categories (p values)	0.15	0.4	0.6	0.5

**Table 3:** Change in the proportion of heavy drinkers during 3 periods surrounding retirement by SES in men (from generalised estimation equations modelling).

	5 to 1 year before		1 year before to 1 year after		1 to 5 years after	
SES	% (95% CI)	p	% (95% CI)	p	% (95% CI]	p
Managers	0.4 (−1.2 to 2.0)	0.6	3.1(1.6 to 4.6)	10−3	−2.0 (−3.6 to −0.4)	0.02
Intermediate	−0.8 (−0.8 to 0.0)	0.1	3.2 (2.4 to 4.0)	10−3	−2.4 (−3.2 to −1.2)	10−3
Clerical workers	−2.6 (−5.6 to 0.4)	0.09	4.6 (1.9 to 7.4)	0.001	−2.8 (−6.0 to 0.0)	0.07
Manual workers	−0.8 (−2.4 to 0.8)	0.3	1.3 (−0.1 to 2.7)	0.07	−0.4 (−2.0 to 0.4)	0.5
Slope difference betweenSES categories (p-values)	0.33		0.069		0.25	

Five years after retirement, there were no longer any differences in heavy drinking between the SES categories, but the overall estimated prevalence of heavy drinking was still quite high, ranging from 15.3% to 16.7% according to the SES category.

### Trajectories of heavy drinking in women

Five years before retirement, the estimated proportion of heavy drinkers differed slightly across SES groups (p = 0.09): 17.3% among managers, 12.0% among intermediate occupations and 10.4% among clerical workers (Table 4). Table 5 shows there was little change in the proportion of heavy drinkers between −5 and −1 year to retirement in any SES group. In the time window from −1 to +1 year after retirement, the estimated percentage of heavy drinkers increased by 6.6% among managers, by 4.3% for intermediate occupations and by 3.3% for clerical workers. The tests for change in slope from −5 to −1 years before retirement, to the slope from −1 to +1 years were statistically significant, except for managers (p = 0.1) (results not shown). There were no differences in the changes (slopes) in the proportion of heavy drinkers between the SES categories. During the period from +1 to +5 years after retirement, there was a decrease in the proportion of heavy drinkers among intermediate and clerical workers, statistically significant in the intermediate SES group (−2.4%), but not among the women managers. Five years after retirement, 24.4% of the managers, 13.5% of the intermediate occupations and 12.6% of the clerical workers were heavy drinkers (table 4), the difference between managers and the other groups being significant p = 0.05 (managers compared to clerical workers: p = 0.01; compared to intermediate occupations: p = 0.03; data not shown).

**Table 4:** Proportion (95% confidence intervals [95%CI]) of heavy drinkers in relation to retirement, by SES in women (from generalised estimation equations modelling).

SES	5 years before	1 year before	1 year after	5 years after
	% (95%CI)	% (95%CI)	% (95%CI)	% (95%CI)
Managers	17.3 (10.2 to 24.4)	16.7 (9.6 to 23.7)	24.4 (16.6 to 32.2)	24.4 (16.0 to 32.8)
Intermediate	12.0 (10.4 to 13.7)	11.6 (9.9 to 13.2)	15.8 (14.0 to 17.7)	13.5 (11.6 to 15.3)
Clerical workers	10.4 (8.5 to 12.4)	10.5 (8.5 to 12.5)	13.8 (11.6 to 15.9)	12.6 (10.4 to 14.8)
Difference between SES categories (p values)	0.09	0.2	0.03	0.05

**Table 5:** Change in the proportion of heavy drinkers during 3 periods surrounding retirement by SES group in women (from generalised estimation equations modelling).

SES	5−1 year before		1 year before −1 year after		1–5 year after	
	% (95%CI)	p	% (95%CI)	p	% (95%CI)	p
Managers	−0.6 (−7.2 to 6.0)	0.8	6.6 (0.1 to 13.1)	0.04	0.0 (−7.6 to 7.6)	1
Intermediate	−0.4 (−2.0 to 1.2)	0.5	4.3 (2.7 to 5.8)	10−3	−2.4 (−4.0 to −0.6)	0.007
Clerical workers	0.0 (−1.6 to 2.0)	0.9	3.3 (1.4 to 5.1)	0.001	−1.2 (−3.2 to 0.8)	0.2
Slope difference betweenSES categories (p-values)	0.8		0.5		0.6	

### Sensitivity analysis for missing data

**Figure 4:**
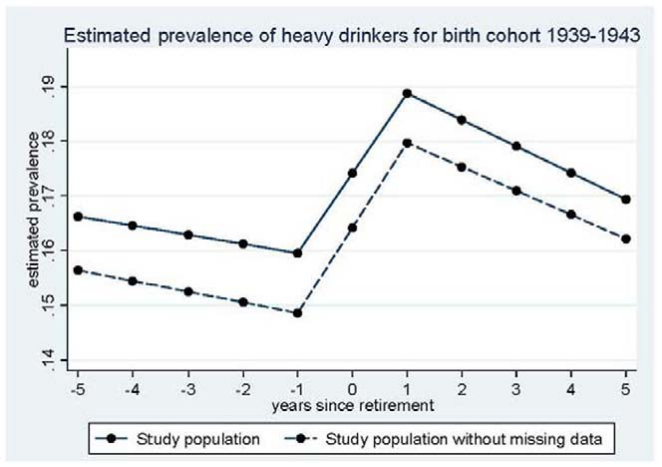
Sensitivity analysis for missing data - Men.

**Figure 5:**
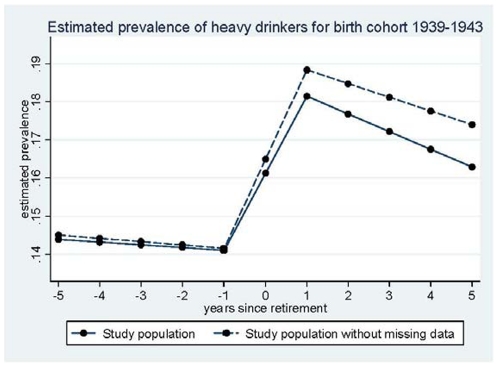
Sensitivity analysis for missing data - Women.

A sensitivity analysis comparing participants with no missing data to the whole study population showed that the pattern of change around the year of retirement differed little between the two groups (Figures 4 and 5).

## Discussion

Longitudinal analysis using repeated measures of alcohol consumption showed an increase in the proportion of heavy drinkers around retirement among both men and women in the Gazel cohort. Among men, this increase was temporary and was followed by a return to the levels observed 5 years before retirement. Among women, the elevated levels of heavy drinking remained unchanged during the entire 5-year post-retirement observation period for managers. Thus these data suggest that retirement is a life transition which may increase the risk of excessive alcohol consumption, temporarily in most people and permanently in the small group of women managers.

A major strength of this study is that it is based on measurements repeated yearly over an extended period of time, from 5 years before to 5 years after retirement. Due to the public sector status of EDF-GDF during the study period, employment was very stable and only 108 participants (0.5%) were lost to follow-up. We had access to comprehensive data on occupational mobility, disability and retirement provided by the company for all Gazel cohort members. Some limitations are noteworthy. Since the data on alcohol consumption were self-reported, there might be some underreporting of the levels of alcohol consumption. However, the fact that confidential questionnaires encourage fuller reports of drinking behavior than face-to-face personal interviews is well-established and [14], [20], [21]. We estimated the weekly number of drinks from the number of days during the past week subjects reported that they drank alcohol and the maximum quantity consumed per day; this could yield to an overestimation of the proportion of heavy workers if levels of drinking vary to a great extent from one day to another. However, this is balanced by the fact that alcohol consumption is usually underreported; moreover, the drinking pattern in France is mainly regular as in other South European countries like Italy where the questionnaire we use in Gazel was developed and validated [14]. It is likely that the way we computed weekly consumption did not provide major overestimation of the proportion of heavy drinkers as it is further suggested by the fact that the rate of heavy drinkers in the Gazel cohort is close to the rate in the French general population measured by other surveys using different tools [22]. Moreover, we were especially interested in trajectories of drinking habits and not on the estimation of the frequency of heavy drinking, and it is likely that the subjects' potential under or over reporting should not have biased the results. Non-response may be a concern, since heavy drinkers are more likely to have missing data [12], [13]. However, less than 10% (1295/14 200) of the participants were excluded from the study due to missing data on alcohol consumption. Sensitivity analyses comparing two different profiles of responders (full data in all years in between −5 to +5 versus all cohort members) showed, as expected, that the proportion of heavy drinkers was higher among the participants with missing data. However, changes in alcohol consumption over time were similar in participants with and without missing observations. Thus, it is unlikely that non-response and loss to follow-up are potential sources of bias for the results regarding the changes in alcohol consumption from pre- to postretirement observed in this study. Finally, although observational data cannot exclude residual confounding and prove causality, our findings strongly suggest an important role of retirement in the modification of alcohol consumption: the prevalence of heavy alcohol consumption is stable in the years preceding retirement but increases sharply at retirement in both sexes and remains elevated among women.

The serial data for this study came from the French national gas and electricity company. Workers employed by the company operate throughout France, both in rural and urban areas, and are employed in a wide range of occupations. Typically, employees have civil servant status that guarantees job stability and opportunities for occupational mobility; employees stay with the company for their whole career, until retirement between the age of 55 and 60, and their pensions (80% of their salary) are paid by the company. It is as yet difficult to assess the extent to which the results observed in this cohort would hold for other working populations, other conditions of employment, or in other cultural settings. Most studies on retirement and alcohol consumption have been conducted in the USA, a country which differs from France in terms of consumption levels and patterns of drinking [5], [23], [24]. In the 50–59 age group of the National Epidemiologic Survey on Alcohol and Related Conditions sample, 98% of women drank less than 13 glasses/week and 93% of men drank less than 20 glasses/week [25]. These figures are considerably lower than those observed in France [24] where alcohol is typically consumed in the form of wine with meals and alcohol is much less often consumed to intoxication, as it is in Anglo-American and Northern European countries. Another point to consider is that the participants in this study were rather young at retirement according to standards in the United States where most of the prior studies were conducted, and continued to have a reasonable income after retirement. These characteristics may have increased the likelihood that the participants were in reasonably good health, and thus might be more likely to be able to continue to consume alcohol without ill effects related to their health status and had sufficient funds to be able to afford alcohol, especially in France where the price of alcoholic beverages is quite low. Patterns of consumption also differ between France and the binge drinking patterns observed in Anglo-American cultural settings and in Northern Europeans where most of the epidemiological evidence used to define low risk levels of consumption comes from. It may be that alcohol consumption declines more steeply with age in a binge drinking culture, especially in this age group since binge drinking habits decrease earlier in life, than in France with more regular drinking with meals. A concern regarding the implications of our findings is that EDF-GDF workers have better pensions than the general French population, potentially allowing them to afford drinking more alcohol than less well-off people. However, one should consider that the price of alcoholic beverages is quite low in France and accessibility is easy. In fact, our data showed that the increase in alcohol consumption was greater among intermediate occupations and clerical workers than for managers in spite of their lower income, which in line with the fact that socioeconomic groups drink more than better-off categories in France, especially among men [22].

The high-resolution data used in this study enabled us to detect complex effect patterns. Probably the most striking result was that the changes in alcohol drinking seem to occur within a short time span around retirement, and that, for most men and women in our study population, they disappeared a few years after retirement. This pattern may explain the inconsistencies in previous studies since longer gaps in data collection mean that transient changes in alcohol consumption are easily missed. Our findings are in agreement with previous studies on retirement and other health-related outcomes within the Gazel cohort, which reported that changes in self-rated health, sleep disturbances, fatigue and depression were particularly apparent during a short time span around retirement [26]–[28]. The design of the Gazel cohort, relying on an annual follow-up over an extended period of time, provided a unique opportunity to detect short-term effects of retirement on alcohol consumption.

Except for men in manual occupations, the increase in heavy drinking around retirement was clear-cut for both sexes. The reasons for the increase remain unclear. Since earlier research in this cohort has shown retirement to have positive effects on health, it seems unlikely that the observed increase in alcohol consumption is a stress-related response [26]–[28]. As has been shown previously, a plausible explanation could be that increased leisure time after retirement provides more opportunities for drinking, and not having to work during the day after may decrease constraints on drinking [29]. Additionally, the festive atmosphere surrounding retirement could lead to increased drinking, which would also explain the transient nature of the increase in some groups. In France, a positive attitude to alcohol drinking is very common and alcohol is an integral part of the general culture: only 20% of the population considers alcohol to be an addictive drug [30].

Among manual workers, the changes in alcohol consumption in relation to retirement were smaller than in other SES groups. Previous work in the Gazel cohort showed sharp social differences in alcohol consumption during working life, the proportion of heavy drinkers being larger among manual workers [31]. One possible explanation for the higher alcohol consumption among manual workers is their difficult working conditions [32]. These findings, in combination, are consistent with the hypothesis that alcohol drinking is associated with social occasions, particularly among people from the higher social categories, while for manual workers alcohol may also be a way to cope with hard working conditions. Relief from work in the form of retirement would decrease the need for alcohol consumption, thereby counteracting the increasing trend in alcohol use in the post-retirement phase seen among higher SES groups. This hypothesis is supported by previous findings from the Gazel study, showing that retirement was associated with a decrease in risky driving behavior, a stronger decrease in the prevalence of suboptimal health, sleep disturbances or fatigue and depression, for those who had had a poor work environment before retirement [26], [28], [33]. Thus, retirement could be perceived as less stressful than working life, especially in this cohort where retirement entails only a relatively small reduction in income [34].

Among women, there was an increase in the proportion of heavy drinkers in each SES category, but the greatest increase was found among managers. This observation is in accordance with the evolution of gendered social roles at work, which is associated with a convergence in the alcohol consumption patterns of men and women, as observed in France, especially among the most affluent social categories [30], [35], [36]. Another possible explanation could be that they have more disposable income than the women of other occupational groups, but this is unlikely to play a major role, since one would expect the same effect among men, which is not the case as detailed above, increase in alcohol consumption being greater for low socioeconomic categories than for managers.

### Implications

In our study, more than 16% of men consumed at least 28 units of alcohol per week five years after retirement. The corresponding proportion among women managers drinking 14 units or more per week was 24.4%. These amounts exceed recommended drinking guidelines for older adults (NIAAA) and are associated with late-life health problems related to drinking [37]. Our findings of increased consumption around retirement, or more worryingly the sustained increase in consumption post-retirement among some groups of women, suggest that information about negative effects of alcohol consumption should be included in pre-retirement planning programs. Preventive measures specifically targeted at the retired population are important since the protective effects of moderate drinking are widely advertised, and individuals may misinterpret information regarding the benefits of alcohol consumption on general health as well as misunderstand the amount and frequency that is beneficial in older people.

The fact that the increase of alcohol consumption seems to be transient for most workers categories should not prevent from targeted interventions, since high levels of drinking may cause harmful events, such as accident: for instance, it was showed in a previous study in Gazel that retirement had no influence on driving while intoxicated [33].

## References

[1] Lopez AD, Mathers CD, Ezzati M, Jamison DT, Murray CJ (2006). Global and regional burden of disease and risk factors, 2001: systematic analysis of population health data.. Lancet.

[2] National Institute on Alcohol Abuse and Alcoholism website.. http://www.niaaa.nih.gov/Publications/AlcoholResearch/.

[3] Bowling A, Dieppe P (2005). What is successful ageing and who should define it?. BMJ.

[4] Britton A, Shipley M, Singh-Manoux A, Marmot MG (2008). Successful aging: the contribution of early-life and midlife risk factors.. J Am Geriatr Soc.

[5] Perreira KM, Sloan FA (2001). Life events and alcohol consumption among mature adults: a longitudinal analysis.. J Stud Alcohol.

[6] Brennan PL, Schutte KK, Moos R (2010). Retired status and older adults' 10-year drinking trajectories.. J Studies Alcohol and Drugs.

[7] Ekert DJ, De Labry LO, Glynn RJ, Davis RW (1989). Change in drinking behaviors with retirement: findings from the Normative Aging Study.. J Stud Alcohol.

[8] Bacharach SB, Bamberger PA, Sonnenstuhl WJ (2004). Retirement, risky alcohol consumption and drinking problem among blue collar workers.. J Stud Alcohol.

[9] Menvielle G, Kunst AE, Stirbu I, Borrell C, Bopp M (2007). Socioeconomic inequalities in alcohol related cancer mortality among men: to what extent do they differ between Western European populations?. Int J Cancer.

[10] Goldberg M, Leclerc A, Bonenfant S, Chastang JF, Schmaus A (2007). Cohort profile: the Gazel Cohort Study.. Int J Epid.

[11] Zins M, Leclerc A, Goldberg M (2009). The French Gazel Cohort Study: 20 years of epidemiologic research.. Advances in Life Course Research.

[12] Goldberg M, Chastang JF, Leclerc A, Zins M, Bonenfant S (2001). Socioeconomic, demographic, occupational and health factors associated with participation in a long-term epidemiologic survey. A prospective study of the French Gazel cohort and its target population.. Am J Epid.

[13] Goldberg M, Chastang JF, Zins M, Niedhammer I, Leclerc A (2006). Health problems were the strongest predictors of attrition during follow-up of the Gazel Cohort.. Journal of Clinical Epidemiology.

[14] Corrao G, Lepore AR, Rapone C, Miccoli C, di Orio F (1991). Reproductibility of an alcohol questionnaire for a case-control study on chronic liver diseases.. Epidemiol Prev.

[15] WHO (2000). World Health Organization. International Guide for Monitoring Alcohol Consumption and Related Harm.

[16] National Institute of Statistics and Economic Studies website.. http://www.insee.fr/en.

[17] Melchior M, Berkman LF, Kawachi I, Krieger N, Zins M (2006). Lifecourse socioeconomic trajectory and premature mortality (35–65) in France: Findings from the GAZEL cohort study.. J Epid Com Health.

[18] Lipsitz SR, Kim K, Zhao L (1994). Analysis of repeated categorical data using generalized estimating equations.. Stat Med.

[19] Harrell FE (2001). Regression Modeling Strategies with Applications to Linear Models, Logistic Regression and Survival Analysis.

[20] Gmel G (2000). The effect of mode of data collection and of non-response on reported alcohol consumption: a split-sample study in Switzerland.. Addiction.

[21] Kraus L, Augustin R (2001). Measuring alcohol consumption and alcohol-related problems: comparison of responses from self-administered questionnaires and telephone interviews.. Addiction.

[22] Guilbert P, Gautier A, INPES (2007). Baromètre Santé 2005 (Health Barometer 2005).. (French Board for Health Education).

[23] Richman AJ, Zlatoper KW, Zackula Ehmke JL, Rospenda JM (2006). Retirement and drinking outcomes: lingering effect of workplace stress?. Addictive Behaviors.

[24] Rehm J, Rehm N, Room R, Monteiro M, Gmel G (2003). The global distribution of average volume of alcohol consumption and patterns of drinking.. Eur Addict Res.

[25] Chan KK, Neighbors C, Gilson M, Larimer ME, Alan Mariatt G (2007). Epidemiological trends in drinking by age and gender: providing normative feedback to adults.. Addict Behav.

[26] Westerlund H, Kivimaki M, Singh-Manoux A, Melchior M, Ferrie JE (2009). Self-rated health before and after retirement in France (Gazel): a cohort study.. Lancet.

[27] Vahtera J, Westerlund H, Hall M, Sjösten N, Kivimäki M (2009). Effect of retirement on sleep disturbances: the Gazel prospective cohort study.. Sleep.

[28] Westerlund H, Vahtera J, Ferrie JE, Singh-Manoux A, Pentti J (2010). Effect of retirement on major chronic conditions and fatigue: French Gazel occupational Cohort Study.. BMJ.

[29] Jokela M, Ferrie JE, Gimeno D, Chandola T, Shipley MJ (2010). From midlife to early old age: health trajectories associated with retirement.. Epidemiology.

[30] Beck F, Legleye S, Spilka S (2008). (Multiple psychoactive substance use (alcohol, tobacco and cannabis) in the French general population in 2005).. Presse Med.

[31] Zins M, Carle F, Bugel I, Leclerc A, Di Orio F (1999). Predictors of change in alcohol consumption among Frenchmen of the Gazel study cohort.. Addiction.

[32] Leclerc A, Zins M, Bugel I, Chastang JF, David S (1994). Consommation de boissons alcoolisées et situation professionnelle dans la Cohorte Gazel (EDF-GDF).. Archives des Maladies Professionnelles.

[33] Bhatti J, Constant A, Salmi LR, Chiron M, Lafont S (2008). Impact of retirement on risky driving behaviors and attitudes toward road safety among a large cohort of French drivers (the Gazel cohort).. Scand J Work Envir and Health.

[34] Bosse R, Aldwin CM, Levenson MR, Workman-Daniels K (1991). How stressful is retirement? Findings from the Normative Aging Study.. J Gerontol.

[35] Wilsnack RW, Kristjanson AF, Wilsnack SC, Crosby RD (2006). Are U.S. women drinking less (or more)? Historical and aging trends, 1981–2001.. J Stud Alcohol.

[36] Lemke S, Schutte KK, Brennan PL, Moos RH (2008). Gender differences in social influences and stressors linked to increase drinking.. J Stud Alcohol Drugs.

[37] Ferreira MP, Weems MKQ (2008). Alcohol Consumption by Aging Adults in the United States: Health Benefits and Detriments.. J Am Diet Assoc.

